# The II Brazilian Guidelines for the pharmacological treatment of patients hospitalized with COVID-19 Joint Guidelines of the *Associação Brasileira de Medicina de Emergência, Associação de Medicina Intensiva Brasileira, Associação Médica Brasileira, Sociedade Brasileira de Angiologia e Cirurgia Vascular, Sociedade Brasileira de Infectologia, Sociedade Brasileira de Pneumologia e Tisiologia and Sociedade Brasileira de Reumatologia*

**DOI:** 10.5935/2965-2774.20230136-en

**Published:** 2023

**Authors:** Maicon Falavigna, Cintia Laura Pereira de Araujo, Alexandre Naime Barbosa, Karlyse Claudino Belli, Verônica Colpani, Felipe Dal-Pizzol, Rosemeri Maurici da Silva, Luciano César Pontes de Azevedo, Maria Beatriz Souza Dias, José Luiz Gomes do Amaral, Gilson Pires Dorneles, Juliana Carvalho Ferreira, Ana Paula da Rocha Freitas, Débora Dalmas Gräf, Hélio Penna Guimarães, Suzana Margareth Ajeje Lobo, Flávia Ribeiro Machado, Michelle Silva Nunes, Maura Salaroli de Oliveira, Suena Medeiros Parahiba, Regis Goulart Rosa, Vania Cristina Canuto Santos, Marcone Lima Sobreira, Viviane Cordeiro Veiga, Ricardo Machado Xavier, Alexandre Prehn Zavascki, Cinara Stein, Carlos Roberto Ribeiro de Carvalho

**Affiliations:** 1 Hospital Moinhos de Vento - Porto Alegre (RS), Brazil; 2 Sociedade Brasileira de Infectologia - São Paulo (SP), Brazil; 3 Universidade do Extremo Sul Catarinense - Criciúma (SC), Brazil; 4 Sociedade Brasileira de Pneumologia e Tisiologia - São Paulo (SP), Brazil; 5 Hospital de Clínicas, Faculdade de Medicina, Universidade de São Paulo - São Paulo (SP), Brazil; 6 Associação Médica Brasileira - São Paulo (SP), Brazil; 7 Associação de Medicina Intensiva Brasileira - São Paulo (SP), Brazil; 8 Associação Brasileira de Medicina de Emergência - São Paulo (SP), Brazil; 9 Ministério da Gestão e da Inovação em Serviços Públicos - Brasília (DF), Brazil; 10 Sociedade Brasileira de Angiologia e de Cirurgia Vascular - São Paulo (SP), Brazil; 11 Sociedade Brasileira de Reumatologia - São Paulo (SP), Brazil

**Keywords:** COVID-19, COVID-19/drug therapy, Coronavirus infections, SARS-CoV-2, Health planning guidelines, Brazil

## Abstract

**Objective:**

To update the recommendations to support decisions regarding the pharmacological treatment of patients hospitalized with COVID-19 in Brazil.

**Methods:**

Experts, including representatives of the Ministry of Health and methodologists, created this guideline. The method used for the rapid development of guidelines was based on the adoption and/or adaptation of existing international guidelines (GRADE ADOLOPMENT) and supported by the e-COVID-19 RecMap platform. The quality of the evidence and the preparation of the recommendations followed the GRADE method.

**Results:**

Twenty-one recommendations were generated, including strong recommendations for the use of corticosteroids in patients using supplemental oxygen and conditional recommendations for the use of tocilizumab and baricitinib for patients on supplemental oxygen or on noninvasive ventilation and anticoagulants to prevent thromboembolism. Due to suspension of use authorization, it was not possible to make recommendations regarding the use of casirivimab + imdevimab. Strong recommendations against the use of azithromycin in patients without suspected bacterial infection, hydroxychloroquine, convalescent plasma, colchicine, and lopinavir + ritonavir and conditional recommendations against the use of ivermectin and remdesivir were made.

**Conclusion:**

New recommendations for the treatment of hospitalized patients with COVID-19 were generated, such as those for tocilizumab and baricitinib. Corticosteroids and prophylaxis for thromboembolism are still recommended, the latter with conditional recommendation. Several drugs were considered ineffective and should not be used to provide the best treatment according to the principles of evidence-based medicine and to promote resource economy.

## INTRODUCTION

The disease caused by severe acute respiratory syndrome coronavirus 2 (SARS-CoV-2), COVID-19, was first identified in Wuhan, China, in December 2019.^(1)^ With the global increase in cases, the World Health Organization (WHO) declared COVID-19 a pandemic on March 11, 2020, requiring global efforts for its prevention and control.^(2)^

Worldwide, as of February 08, 2023, the WHO has reported more than 755 million confirmed cases and more than 6.8 million deaths due to COVID-19.^(3)^ In Brazil, as of February 08, 2023, 36,907,890 COVID-19 cases and 697,583 deaths due to COVID-19 have been confirmed.^(4)^ In most cases, people with COVID-19 experience mild clinical manifestations of the disease, such as fever, dry cough and fatigue, and the disease resolves in a self-limiting manner. However, in approximately 14% of cases, COVID-19 can progress to severe disease, which may require oxygen therapy or hospitalization. Patients with COVID-19 who require intensive care unit (ICU) admission for acute respiratory failure due to viral pneumonia usually exhibit an increased respiratory rate and hypoxemia, which may progress to sepsis, septic shock and multiple organ failure, including acute kidney injury and cardiac injury.^(5)^

Vaccination has an impact on hospitalizations and deaths. However, even among vaccinated individuals, uncertainties remain about the duration of protection and effectiveness of current vaccines, as well as about the efficacy of existing treatments for COVID-19 against emerging SARS-CoV-2 variants and subvariants.^(5)^

Furthermore, since the publication of the first version of this guideline in June 2022,^(6)^ there have been advances in knowledge of the pharmacological treatment of patients hospitalized with COVID-19 and changes in the regulations on the use of medication in Brazil, such as the suspension of the use authorization of casirivimab + imdevimab, the authorization to use baricitinib and new evidence on the use of tocilizumab. Furthermore, the Pan-American Guidelines for the Treatment of SARS-CoV-2/COVID-19^(7)^ were recently published and include recommendations for the use of remdesivir, baricitinib and tocilizumab for the treatment of patients hospitalized with COVID-19. Therefore, it is necessary to update the guidelines in the Brazilian context.

This updated guideline for the pharmacological treatment of patients hospitalized with COVID-19 was developed by the Ministry of Health in conjunction with seven medical specialty societies. The objective of the document was to provide uniformity in the therapeutic indications for patients with COVID-19 in the context of hospital treatment and to guide therapeutic interventions, making use of the best evidence available at the time of its elaboration.

## METHODS

The update of the previous guidelines^(6)^ followed the method for developing rapid guidelines based on the adoption and/or adaptation of recommendations in existing international guidelines, which were identified through the e-COVID-19 RecMap platform and additional searches for primary studies and the addition of new recommendations when necessary (GRADE ADOLOPMENT).^(8,9)^

The target audience was composed of health professionals involved in the care of adult patients hospitalized with COVID-19, especially intensivists, internists, emergency physicians, infectious disease specialists, pulmonologists and clinical pharmacists. Nonhospitalized patients with COVID-19 and pregnant and postpartum women were not target population of these guidelines. Likewise, this document did not evaluate interventions in primary health care or specialized outpatient care.

### Guideline development group

The group involved in the development of this guideline was composed of a panel of experts under the management of the Department of Management and Incorporation of Technologies and Innovation in Health (DGITIS - *Departamento de Gestão e Incorporação de Tecnologias e Inovação em Saúde*) of the Secretariat of Science, Technology and Strategic Inputs (SCTIE - *Secretaria de Ciência, Tecnologia e Insumos Estratégicos*) of the Ministry of Health. This update was prepared by 13 experts (six methodologists and seven members of a panel of experts) and was reviewed and updated between June and November 2022. The panel of experts included intensive care physicians, internists and emergency physicians, vascular and endovascular surgeons, infectious disease specialists, rheumatologists, pulmonologists, pharmacists, representatives of the Ministry of Health, professionals from universities and hospitals of excellence in Brazil, and methodologists. The following medical societies participated in the development of this guideline and endorsed its recommendations: *Associação Brasileira de Medicina de Emergência* (ABRAMEDE), *Associação de Medicina Intensiva Brasileira* (AMIB), *Associação Médica Brasileira* (AMB), *Sociedade Brasileira de Angiologia e Cirurgia Vascular* (SBACV), *Sociedade Brasileira de Infectologia* (SBI), *Sociedade Brasileira de Pneumologia e Tisiologia* (SBPT), and *Sociedade Brasileira de Reumatologia* (SBR).

The guidelines for pharmacological treatment of patients hospitalized with COVID-19, prepared by the panel of experts, were reviewed and updated between June and November 2022. In this update, the management committee organized one virtual meeting with the experts by videoconference to develop and discuss the guidelines based on the new evidence available on the drug treatment of patients with COVID-19, adapted to the national context. The members of the management committee and the methodologists did not interfere in the experts’ preparation of the guidelines. The list of participants, their role in the guidelines and the declaration of conflicts of interest are presented in the Supplementary Material.

### Research questions

The technologies evaluated in eight international guidelines^(5,10-16)^ for the treatment of COVID-19 were reviewed to identify the clinical issues of interest, according to the method described in the first guideline.^(6)^ Thirteen clinical questions were prepared according to the PICO method (population, intervention, comparator and outcome) to consider the following therapies: anticoagulants, antimicrobials, azithromycin, baricitinib, casirivimab + imdevimab, chloroquine or hydroxychloroquine, colchicine, corticosteroids, ivermectin, lopinavir + ritonavir, convalescent plasma, remdesivir, and tocilizumab.

### Search and synthesis of evidence

In this update, the source documents for identifying evidence were existing guidelines, with complementary systematic reviews carried out when needed to include evidence not covered in the selected guidelines. The recommendations, evidence profiles, and Grading of Recommendations Assessment, Development and Evaluation (GRADE) domains were extracted from the evidence tables for decision-making using the e-COVID-19 RecMap platform. The original documents were evaluated when necessary.^(8,9)^

The following guidelines were used in the adaptation process:

- World Health Organization (WHO): Therapeutics and COVID-19 - Living Guideline (April 2022).^(5)^
- Australian National COVID-19 Clinical Evidence Taskforce: Caring for people with COVID-19 - Supporting Australia’s healthcare professionals with continually updated, evidence-based clinical guidelines (June 2022).^(12)^
- Infectious Diseases Society of America (IDSA): Infectious Diseases Society of America Guidelines on the Treatment and Management of Patients with COVID-19 (May 2022).^(11)^
- AMIB, SBI, and SBPT: *Diretrizes para o tratamento farmacológico da COVID-19. Consenso da Associação de Medicina Intensiva Brasileira, da Sociedade Brasileira de Infectologia e da Sociedade Brasileira de Pneumologia e Tisiologia* (March 2022).^(12)^
- National Institute for Health and Care Excellence (NICE): COVID-19 rapid guideline: managing COVID-19 (May 2022).^(13)^
- National Institutes of Health (NIH): COVID-19 Treatment Guideline (May 2022).^(14)^
- Society of Critical Care Medicine (SCCM)/Surviving Sepsis Campaign (SCC): Surviving Sepsis Campaign Guidelines on the Management of Adults With Coronavirus Disease 2019 (COVID-19) in the ICU: First Update (March 2021).^(15)^
- European Respiratory Society living guideline (ERS): Management of hospitalised adults with coronavirus disease 2019 (COVID-19): a European Respiratory Society living guideline (June 2022).^(16)^
- American Society of Hematology (ASH): ASH Guidelines on Use of Anticoagulation in Patients with COVID-19 (May 2022).^(17)^
- European League Against Rheumatism (EULAR): EULAR points to consider on pathophysiology and use of immunomodulatory therapies in COVID-19 (January 2022).^(18)^

### Assessment of the certainty of evidence and the development of recommendations

The GRADE system was used to evaluate the certainty of the evidence. We adopted the GRADE evidence profiles presented by the guidelines that most recently conducted an evidence search that answered the research questions of interest. When it was necessary to update information, a systematic review of the literature was performed. Evidence from preprints and press releases was considered a qualitative factor in decision-making and did not modify the level of evidence evaluated by the original documents. According to the GRADE methodology, recommendations can be strong or conditional (weak) for or against an intervention. Certainty of evidence and strength of recommendation according to the GRADE system were previously reported in the first Guidelines published in June 2022.^(6)^

In developing the recommendations, the evidence of benefits and risks, the certainty of evidence, the costs and use of resources, acceptance by professionals and other barriers to implementation were considered. Additional statements about the recommendations, such as potential exceptions to the proposed behaviors or clarifications, were documented throughout the text. The direction and strength of the recommendations, as well as their wording, were determined during the meetings at which the recommendations were prepared.

### Population of interest

The target population of the recommendations was adult hospitalized patients with a diagnosis or suspicion of COVID-19. Nonhospitalized patients with COVID-19 and pregnant and postpartum women were not targets of this guideline.

## RESULTS

**Figure 1:**
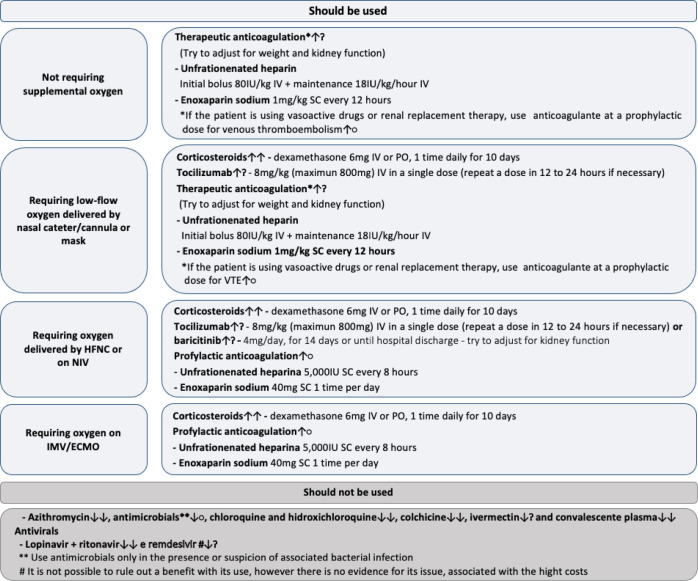
Recommendations for the pharmacological treatment of patients hospitalized with COVID-19.

Twenty-one recommendations were made. Recommendations for the use of baricitinib were included, recommendations on the use of anticoagulants and tocilizumab were changed, and the recommendation on the use of casirivimab + imdevimab was removed. The other recommendations were not changed. The recommendations are summarized in table 1 and in figure 1. Below, we present the recommendations, the rationale for the decisions and, when relevant, considerations for implementation. Detailed information on the evidence supporting each recommendation is presented in the Supplementary Material.

**Table 1:** Summary of recommendations

Medication	Recommendation
**Anticoagulants**	**Recommendation 1.1** - We recommend the use of anticoagulants at prophylactic doses for VTE in critically ill patients (those using vasoactive drugs and those undergoing renal replacement therapy, HFNC, NIV or IMV) with COVID-19 (nongraded recommendation)
	**Recommendation 1.2** - We suggest against the use intermediate doses or therapeutic anticoagulation in critically ill COVID-19 patients (those using vasoactive drugs or undergoing renal replacement therapy, HFNC, NIV or IMV) without evidence of thromboembolism (conditional recommendation, very low certainty of evidence)
	**Recommendation 1.3** - We suggest the use of heparin or enoxaparin in therapeutic doses in noncritical patients (those with no need for vasoactive drugs, renal replacement therapy, HFNC, NIV or IMV) hospitalized with COVID-19 (conditional recommendation, very low certainty of evidence)
**Antimicrobials**	**Recommendation 2.1** - We recommend against the use of antimicrobials in patients with COVID-19 without suspected bacterial infection (nongraded recommendation)
**Azithromycin**	**Recommendation 3.1** - We recommend against the use of azithromycin, with or without chloroquine or hydroxychloroquine, in patients hospitalized with COVID-19 (strong recommendation, moderate certainty of evidence)
**Baricitinib**	**Recommendation 4.1** - We suggest against the use of baricitinib in patients hospitalized with COVID-19 who are not on supplemental oxygen (conditional recommendation, low certainty of evidence)
	**Recommendation 4.2** - We suggest against the use of baricitinib in patients hospitalized with COVID-19 who are on low-flow supplemental oxygen (conditional recommendation, moderate certainty of evidence)
	**Recommendation 4.3** - We suggest the use of baricitinib in hospitalized patients with COVID-19 who are on HFNC or NIV (conditional recommendation, moderate certainty of evidence)
**Casirivimab + imdevimab**	**Recommendation 5.1** - It is not possible to issue a recommendation for its use at the moment (November 2022) in view of the suspension of authorization for emergency use by Anvisa
**Chloroquine or hydroxychloroquine**	**Recommendation 6.1** - We recommend against the use of chloroquine or hydroxychloroquine in patients hospitalized with COVID-19 (strong recommendation, moderate certainty of evidence)
**Colchicine**	**Recommendation 7.1** - We recommend against the use of colchicine in patients hospitalized with COVID-19 (strong recommendation, moderate certainty of evidence)
**Corticosteroids**	**Recommendation 8.1** - We recommend the use of 6mg of dexamethasone intravenously or orally once daily for 10 days in patients who are hospitalized with COVID-19 and using supplemental oxygen (strong recommendation, moderate certainty of evidence)
	**Recommendation 8.2** - We suggest against the use of corticosteroids in patients hospitalized with COVID-19 who are not using supplemental oxygen (conditional recommendation, low certainty of evidence)
**Ivermectin**	**Recommendation 9.1** - We suggest against the used of ivermectin in patients hospitalized with COVID-19 (conditional recommendation, very low certainty of evidence)
**Lopinavir + ritonavir**	**Recommendation 10.1** - We recommend against the use of lopinavir + ritonavir in patients hospitalized with COVID-19 (strong recommendation, moderate certainty of evidence)
**Convalescent plasma**	**Recommendation 11.1** - We recommend against the use of convalescent plasma in patients hospitalized with COVID-19 (strong recommendation, moderate certainty of evidence)
**Remdesivir**	**Recommendation 12.1** - We suggest against the used of remdesivir in patients hospitalized with COVID-19 (conditional recommendation, low certainty of evidence).
**Tocilizumab**	**Recommendation 13.1** - Due to the lack of evidence of the use of tocilizumab in hospitalized patients with COVID-19 who are not on supplemental oxygen, it is not possible to make a recommendation (no recommendation)
	**Recommendation 13.2** - We suggest the use of tocilizumab in hospitalized patients with COVID-19 who are on low-flow supplemental oxygen (conditional recommendation, moderate certainty of evidence)
	**Recommendation 13.3** - We suggest the use of tocilizumab in hospitalized patients with COVID-19 who are on HFNC or NIV (conditional recommendation, moderate certainty of evidence)
	**Recommendation 13.4** - We suggest against the used of tocilizumab in hospitalized patients with COVID-19 who are on IMV or ECMO (conditional recommendation, low certainty of evidence)

VTE - venous thromboembolism; HFNC - high-flow nasal cannula; NIV - noninvasive ventilation; IMV - invasive mechanical ventilation; Anvisa - *Agência Nacional de Vigilância Sanitária*; ECMO - extracorporeal membrane oxygenation.

IV - intravenously; SC - subcutaneous; PO - orally; VTE - venous thromboembolism; HFNC - high-flow nasal cannula; NIV - noninvasive ventilation; IMV - invasive mechanical ventilation; ↑↑ - strong recommendation in favor; ↓↓ - strong recommendation against; ↑? - conditional recommendation in favor; ↓? - conditional recommendation against; ↑○ - nongraded recommendation in favor; ↓○ - nongraded recommendation against.

### Anticoagulants

**Recommendation 1.1 -** We recommend the use of anticoagulants at prophylactic doses for venous thromboembolism (VTE) in critically ill patients (those using vasoactive drugs and those undergoing renal replacement therapy, high-flow nasal cannula - HFNC, noninvasive ventilation - NIV, or invasive mechanical ventilation - IMV) with COVID-19 (nongraded recommendation).

**Recommendation 1.2 -** We suggest against the use of intermediate doses or therapeutic anticoagulation in critically ill COVID-19 patients (those using vasoactive drugs or undergoing renal replacement therapy, HFNC, NIV or IMV) without evidence of thromboembolism (conditional recommendation, very low certainty of evidence).

**Recommendation 1.3 -** We suggest the use of heparin or enoxaparin in therapeutic doses for noncritical patients (those with no need for vasoactive drugs, renal replacement therapy, HFNC, NIV or IMV) hospitalized with COVID-19 (conditional recommendation, very low certainty of evidence).

*(These recommendations changed in the update.*)

**Justification for the recommendation -** The panel of experts considered that there is no benefit from the use of anticoagulants at intermediate or therapeutic doses in critically ill patients with COVID-19. Additionally, anticoagulation is associated with an increased risk of bleeding events and should be avoided in this population. There is a potential benefit from the use of heparin or enoxaparin at therapeutic doses in noncritical patients, and the same effect was not observed for oral anticoagulants (rivaroxaban).

**General and implementation considerations -** In noncritical hospitalized patients with COVID-19 (i.e., those who do not need vasoactive drugs, renal replacement therapy, HFNC, NIV or IMV), therapeutic anticoagulation with unfractionated heparin or enoxaparin may be used according to the individual’s risk of bleeding. Rivaroxaban was not effective in the treatment of hospitalized patients with COVID-19 and was associated with a greater potential number of adverse events.^(19)^

Prophylaxis for VTE should be performed, preferably with unfractionated heparin, although enoxaparin or fondaparinux may be used alternatively. The suggested dosage is shown in table 2. The preference for unfractionated heparin over enoxaparin is based on lower costs and greater availability of the former at the time the recommendation was drafted; however, availability may vary over time and among institutions.

**Table 2:** Dosages of anticoagulant drugs

Medication	Patient group	Dose
**Unfractionated heparin**	Standard dose	5,000 IU subcutaneously every 8 hours
	BMI > 40kg/m^2^	10,000 IU every 12 hours
	Renal insufficiency (CrCl < 30mL/minute)	5,000 IU every 12 hours
**Enoxaparin**	Up to 80 kg	40mg once a day
	Between 80 and 120kg	60mg once a day
	Over 120kg	40mg every 12 hours
	BMI > 50kg/m^2^	60mg every 12 hours
	CrCl < 3mL/minute	Do not use
**Fondaparinux**	Standard dose	2.5mg once a day
	Renal insufficiency (CrCl 20 - 30mL/minute)	2.5mg every 48 hours
	Renal insufficiency (CrCl < 20mL/minute)	Do not use

BMI - body mass index; CrCl - creatinine clearance.

The definition of preferential alternatives can be customized based on the particularities of each institution. Enoxaparin and fondaparinux appear to have similar results; however, enoxaparin has the advantage of a greater number of studies and more experience with its use. Fondaparinux is indicated in patients with suspected or diagnosed heparin-induced thrombocytopenia and may also be used preferentially in patients with thrombocytopenia due to other etiologies. Prophylaxis is contraindicated in patients with platelet counts < 30,000 platelets per mm^3^.

There is no indication for the routine use of anticoagulants post-discharge for COVID-19. The indication for the use of anticoagulants after discharge should follow the same criteria applied for non-COVID-19 patients according to institutional protocols, and instruments such as the Padua score and IMPROVE may be used as support.^(20-22)^ Anticoagulation therapy should be used for patients with specific clinical indications (e.g., atrial fibrillation and VTE) according to their baseline condition.

### Antimicrobials

**Recommendation 2.1 -** We recommend against the use of antimicrobials in patients with COVID-19 without suspected bacterial infection (nongraded recommendation). *(This recommendation did not change.)*

**Justification for the recommendation -** The panel of experts determined that there is no basis for the routine use of antimicrobials in patients with COVID-19 without suspected associated bacterial infection, since coinfection is uncommon.^(23)^

**General and implementation considerations -** Patients with suspected sepsis on admission who do not have a definitive diagnosis of COVID-19 should be managed according to the institutional protocol for sepsis.

Patients with COVID-19 who, on hospital admission, have a potential bacterial focus of infection (e.g., pulmonary radiological consolidation, leukocytosis in the absence of corticosteroid use, purulent secretions) are potential candidates for the empirical use of antimicrobials. The initiation of antimicrobial use should be based on clinical judgment, patient risk factors and local epidemiology. Bacterial cultures (blood culture and culture of the suspected site) should be collected prior to the initiation of antimicrobials. Empirical therapy should be based on guidelines from the local hospital infection control service and/or institutional protocols for the use of antimicrobials. Daily reassessments should be performed to determine the need for de-escalation or suspension of antimicrobial therapy. A high level of suspicion of health care-related infections, such as MV-associated pneumonia, urinary tract infection, and catheter-associated bloodstream infection, should be maintained.

### Azithromycin

**Recommendation 3.1 -** We recommend against the use of azithromycin, with or without chloroquine or hydroxychloroquine, in patients hospitalized with COVID-19 (strong recommendation, moderate certainty of evidence).

*(This recommendation did not change.*)

**Justification for the recommendation -** The panel of experts considered that the evidence shows no benefit from the use of azithromycin in patients hospitalized with COVID-19.^(24-28)^ The drug was not recommended by any of the identified guidelines.

**General and implementation considerations -** Azithromycin can be used in cases of suspected or confirmed bacterial infection, according to the guidelines of the local hospital infection control service and/or institutional protocols for the use of antimicrobials.

### Baricitinib

**Recommendation 4.1 -** We suggest against the use of baricitinib in patients hospitalized with COVID-19 who are not on supplemental oxygen (conditional recommendation, low certainty of evidence).

**Recommendation 4.2 -** We suggest against the use of baricitinib in patients hospitalized with COVID-19 who are on low-flow supplemental oxygen (conditional recommendation, moderate certainty of evidence).

**Recommendation 4.3 -** We suggest the use of baricitinib in hospitalized patients with COVID-19 who are on HFNC or NIV (conditional recommendation, moderate certainty of evidence).

*(These recommendations were not included in the previous document since health technology had not been prioritized)*.

**Justification for the recommendation -** The panel of experts considered that the evidence shows benefit from the use of baricitinib in patients hospitalized with COVID-19 who are on high-flow supplemental oxygen or on NIV. The available evidence suggests an uncertain benefit in patients who do not need supplemental oxygen and who are on low-flow supplemental oxygen, and tocilizumab should be prioritized. These recommendations are in line with the recommendations of the identified guidelines.

**General and implementation considerations -** Studies show the potential benefit of using baricitinib in patients on IMV or extracorporeal membrane oxygenation (ECMO); however, the drug has not been approved by Brazilian regulatory authorities for use in this population, and its incorporation has not been evaluated by the *Comissão Nacional de Incorporação de Tecnologias* (Conitec) in the Sistema Único de Saúde (SUS).

In the population on supplemental oxygen with HFNC or NIV, tocilizumab or baricitinib can be used, as the drugs have similar effectiveness. There is no indication for the combination of baricitinib and tocilizumab.

In patients with renal insufficiency, it is necessary to adjust the dose according to the estimated glomerular filtration rate (eGFR). The recommended dose of baricitinib in patients with an eGFR between 30 and 60mL/minute/1.73 m^2^ is 2mg once daily. Baricitinib is not recommended for use in patients with an estimated eGFR < 30mL/minute/1.73m^2^.

No dose adjustment is required in patients with mild or moderate hepatic impairment. Baricitinib has not been studied in patients with severe hepatic impairment for COVID-19 indication and is therefore not recommended for these patients.

Initiating or discontinuing baricitinib treatment should be avoided in patients with an absolute lymphocyte count < 500 cells/mm^3^.

Attention should be given to the presence of latent infections such as tuberculosis and parasitic infections, in which the use of baricitinib can promote reactivation, especially in critically ill patients already using corticosteroids.

### Casirivimab + imdevimab

**Recommendation 5.1 -** During the update, it was not possible to issue a recommendation for the use of this treatment due to the suspension of the *Agência Nacional de Vigilância Sanitária* (Anvisa) authorization for emergency use.

**Justification for the recommendation -** In the general population of hospitalized patients, monoclonal antibodies do not reduce clinical events. However, two studies showed potential benefit with casirivimab + imdevimab in a subgroup analysis evaluating seronegative patients (no antibodies or low antibody titers for COVID-19). Despite this potential benefit, considering the unavailability of antibody testing in the SUS and the fact that the studies were developed with variants different from the circulating variantsat the time of recommendation, the panel of experts suggests against the routine use of monoclonal antibodies in hospitalized patients with COVID-19.

**General and implementation considerations -** In addition to casirivimab + imdevimab, other monoclonal antibodies are being studied for use in COVID-19 (bamlanivimab and etesevimab); however, they have no documented benefit in this population and do not have a current drug registration in Brazil. The inclusion of hospitalized patients in clinical trials evaluating these drugs is encouraged.

### Chloroquine or hydroxychloroquine

**Recommendation 6.1 -** We recommend against the use of chloroquine or hydroxychloroquine in patients hospitalized with COVID-19 (strong recommendation, moderate certainty of evidence).

*(This recommendation did not change.*)

**Justification for the recommendation -** The panel of experts considered that the evidence shows no benefit from the use of hydroxychloroquine or chloroquine in patients hospitalized with COVID-19.^(25-31)^ The drugs were not recommended by any of the identified guidelines.

**General and implementation considerations -** Chloroquine and hydroxychloroquine should not be used, regardless of the route of administration (oral, inhaled or others). Patients who use chloroquine or hydroxychloroquine due to other health conditions (e.g., rheumatic diseases and malaria) should continue to use them.

### Colchicine

**Recommendation 7.1 -** We recommend against the use of colchicine in patients hospitalized with COVID-19 (strong recommendation, moderate certainty of evidence).

*(This recommendation did not change.*)

Justification for the recommendation - The panel of experts considered that, according to the available evidence, colchicine is not effective in the treatment of hospitalized patients with COVID-19 and is therefore not recommended.^(32-34)^

### Corticosteroids

**Recommendation 8.1 -** We recommend the use of 6mg dexamethasone intravenously (IV) or orally (PO) once daily for 10 days in patients who are hospitalized with COVID-19 and using supplemental oxygen (strong recommendation, moderate certainty of evidence).

**Recommendation 8.2 -** We suggest against the use of corticosteroids in patients hospitalized with COVID-19 who are not using supplemental oxygen (conditional recommendation, low certainty of evidence).

*(This recommendation did not change.*)

**Justification for the recommendation -** The panel of experts considered that there is an important benefit gained from the use of corticosteroids in patients hospitalized with COVID-19 who are using oxygen.^(35,36)^ Along with the proven benefit, which has a moderate certainty of evidence, the drug is well tolerated, widely available and inexpensive, which leads to a strong recommendation in favor of its use in this population. The available evidence suggests a lack of benefit in patients who do not require supplemental oxygen.

**General and implementation considerations -** The preferred drug for use is dexamethasone, as used in the RECOVERY study.^(35)^ Alternatively, if dexamethasone is not available, hydrocortisone can be used at a dose of 50mg IV every 6 hours, or methylprednisolone can be used at a dose of 40mg IV per day. These recommendations took into account that for COVID-19, hydrocortisone and methylprednisolone were the most studied corticosteroids after dexamethasone.^(36)^ Nevertheless, it is important to point out that dexamethasone is the first option, and methylprednisolone and hydrocortisone should only be used when dexamethasone is not available. Other corticosteroids can be used at equivalent doses, such as prednisone 40mg once a day PO. Corticosteroids should not be used in patients who do not require supplemental oxygen.

The use of corticosteroids as recommended (at low doses, limited to 10 days) may be abruptly discontinued, and gradual withdrawal is not necessary. There is also no need to continue treatment after discharge. Oral corticosteroids should be used only in patients with a patent enteral route and may be administered with food. If there is no certainty regarding the suitability of the enteral route (e.g., in a critically ill patient), intravenous administration should be used whenever possible. There is uncertainty regarding the optimal dose for patients on MV. In patients with acute respiratory distress syndrome, evidence suggests that a higher dose (12mg) appears to be more beneficial and may be considered an option.^(37-39)^ Thus, higher doses, limited to 20mg per day of dexamethasone or 100mg per day of methylprednisolone, may be used. There is no evidence of benefit for the use of corticosteroid pulse therapy in patients with COVID-19; the effects of immunosuppression on disease progression are not known, and an increased risk of associated infections is expected.

Patients with other indications for corticosteroids (for example, exacerbated asthma or chronic obstructive pulmonary disease, previous use due to rheumatic diseases, pulmonary maturation in pregnant women) should receive them according to their clinical indication.

It is not possible to make recommendations regarding the replacement of dexamethasone with hydrocortisone in patients with COVID-19 and septic shock, as both alternatives are valid at the established doses; however, the two should not be used concomitantly.

### Ivermectin

**Recommendation 9.1 -** We suggest against the use of ivermectin in patients hospitalized with COVID-19 (conditional recommendation, very low certainty of evidence).

*(This recommendation did not change.*)

Justification for the recommendation - The panel of experts considered that there are no studies that support the use of ivermectin in hospitalized patients with COVID-19, and its use should be restricted to clinical studies.

### Lopinavir + ritonavir

**Recommendation 10.1 -** We recommend against the use of lopinavir + ritonavir in patients hospitalized with COVID-19 (strong recommendation, moderate certainty of evidence).

*(This recommendation did not change.*)

**Justification for the recommendation -** The panel of experts considered that, according to the available evidence, treatment with lopinavir + ritonavir is not effective in the treatment of hospitalized patients with COVID-19 and therefore is not recommended.^(40-42)^

### Convalescent plasma

**Recommendation 11.1 -** We recommend against the use of convalescent plasma in patients hospitalized with COVID-19 (strong recommendation, moderate certainty of evidence).

*(This recommendation did not change.*)

**Justification for the recommendation -** The panel of experts considered that, according to the available evidence, convalescent plasma is not effective in the treatment of hospitalized patients with COVID-19 and is therefore not recommended.^(43-51)^

### Remdesivir

**Recommendation 12.1 -** We suggest against the use of remdesivir in patients hospitalized with COVID-19 (conditional recommendation, low certainty of evidence).

*(This recommendation did not change.*)

**Justification for the recommendation -** Although no reduction in mortality was observed in the general population, a reduction in mortality was observed in the population using low-flow oxygen in the SOLIDARITY study and in the ACTT-1 study.^(52,53)^ The study group considered that there are uncertainties about the magnitude of the benefit in the use of remdesivir; therefore, there is no justification for its routine use in hospitalized patients with COVID-19.(52,53) These uncertainties regarding the clinical benefit, along with the high cost, justify the conditional recommendation against the use of remdesivir at this time.

**General and implementation considerations -** There was a reduction in the time to recovery among patients using low-flow oxygen in a clinical trial. However, there are uncertainties about this benefit and its clinical significance, not justifying its routine use even in this group of patients.

Although no reduction in mortality was observed in the general population, a reduction in mortality was observed in the population using low-flow oxygen in the SOLIDARITY study (HR 0.87; 95%CI 0.76 to 0.98),^(52)^ and in the ACTT-1 study (HR 0.30; 95%CI 0.14 to 0.64).^(53)^ Thus, it is possible that there is at least a marginal benefit with the use of remdesivir, especially in the group of patients using low-flow oxygen.

The study group considered that, despite the possibility of benefit in the group using low-flow oxygen, there are uncertainties about the magnitude of benefit; therefore, there is no justification for its routine use in patients hospitalized with COVID-19. These uncertainties about the relevance of the clinical benefit, associated with the high cost, and its nonincorporation into the SUS after evaluation by Conitec (Ordinance SCTIE/MS n° 60/2021; Recommendation Report n° 655 of August/2021) justify the conditional recommendation against using remdesivir at this time.

### Tocilizumab

**Recommendation 13.1 -** Due to the lack of evidence of the use of tocilizumab in hospitalized patients with COVID-19 who are not on supplemental oxygen, it is not possible to make a recommendation (no recommendation).

**Recommendation 13.2 -** We suggest the use of tocilizumab for hospitalized patients with COVID-19 who are on low-flow supplemental oxygen (conditional recommendation, moderate certainty of evidence).

**Recommendation 13.3 -** We suggest the use of tocilizumab for hospitalized patients with COVID-19 who are on HFNC or NIV (conditional recommendation, moderate certainty of evidence).

**Recommendation 13.4 -** We suggest against the use of tocilizumab in hospitalized patients with COVID-19 who are on IMV or ECMO (conditional recommendation, low certainty of evidence).

*(These recommendations changed in the update.*)

Justification for the recommendation - The panel understands that there is benefit from the use of tocilizumab in patients hospitalized with COVID-19 who are on supplemental oxygen but not on invasive mechanical ventilation or ECMO.^(54,55)^

**General and implementation considerations:** To date, studies have not shown an explicit benefit for patients not using oxygen or using IMV or ECMO.^(55)^ Attention should be given to the presence of latent infections, such as tuberculosis and parasitic infections, for which tocilizumab can promote reactivation, especially in critically ill patients already using corticosteroids. Tocilizumab should not be used in patients with the presence or suspicion of associated bacterial infections. Tocilizumab should be used with caution in immunosuppressed patients. The drug should not be used in patients with neutropenia (< 500 cells), thrombocytopenia (< 50,000) or aminotransferase levels five times above the normal range.

Tocilizumab should be used at a single dose of 8mg/kg IV, with a maximum dose of 800mg. If there is no improvement in 12 to 24 hours, a second dose should be administered. Doses greater than 800mg per infusion are not recommended in patients with COVID-19. If used, tocilizumab should always be accompanied by corticosteroids, with dexamethasone at 6mg administered IV or PO being the recommended regimen.

Baricitinib and tocilizumab are similarly effective in the HFNC or NIV population and can be used according to the choice criteria of each health institution. Tocilizumab should not be associated with the use of baricitinib. If used, it should always be accompanied by corticosteroids, the recommended regimen being 6mg of dexamethasone IV or PO.^(55)^ The manufacturer’s package insert indicates that there is no need for dose adjustment for patients with mild or moderate renal impairment. However, in patients with CrCl < 30mL/minute, drug clearance may be impaired due to its molecular weight, which requires greater vigilance regarding potential adverse effects.

In obese patients, the maximum dose of 800mg achieved the target area under the curve (AUC) and trough concentration in all weight strata, including the highest (160kg). However, more research is needed to assess whether higher doses are needed in patients weighing more than 160kg.^(56)^

The risk-benefit ratio of the use of tocilizumab should be evaluated, especially in patients using low-flow oxygen, for whom the absolute benefit may be limited and the risks may outweigh the benefits.

## DISCUSSION

In this guideline update, which was developed by a panel of experts composed of representatives of medical societies and the Ministry of Health, 21 recommendations were elaborated, including the new recommendation on baricitinib for hospitalized patients with COVID-19 who are on HFNC or NIV, a change in the recommendation for treatment with tocilizumab in hospitalized patients with COVID-19 on supplemental oxygen or NIV, and a change in the recommendation for the use of anticoagulants at prophylactic doses for thromboembolism. The recommendation for treatment with corticosteroids for patients using supplemental oxygen did not change from the last guideline.

During epidemics, when there are no clinical treatments with consolidated effectiveness, there is a tendency to use drugs based on the results of preclinical studies or observational studies with important limitations.^(11)^ Experience from epidemics has shown that these interventions may have a much lower benefit than expected, as was the case for oseltamivir during the swine flu epidemic in 2009. During the Ebola epidemic in 2014, several interventions were tested, including chloroquine, hydroxychloroquine, favipiravir immunobiologicals and convalescent plasma, none of which showed proof of effectiveness or safety.^(57)^ In such situations where drug treatment is not consolidated, providing proper ICU care can improve survival.^(58)^

The understanding of SARS-CoV-2 infection and its treatment has evolved considerably over the past 3 years as a result of the collaborative efforts of several countries and research groups, which have developed randomized clinical studies to evaluate potential candidates for the treatment of COVID-19. Among them, the RECOVERY, REMAP-CAP, SOLIDARITY, and in Brazil, COALIZÃO studies are noteworthy. As a result of these initiatives, some therapies with potential benefit, such as corticosteroids, tocilizumab, and baricitinib,^(35,55)^ were identified. Moreover, several ineffective therapies were discarded to promote safe and evidence-based treatment for the population and to promote the rational allocation of resources, such as azithromycin, chloroquine or hydroxychloroquine, colchicine, ivermectin, lopinavir + ritonavir and convalescent plasma. Although some marginal benefit can be obtained with the use of remdesivir, its high cost does not justify its routine use.

Regarding costs, in terms of public health, it is important to note that in an epidemic scenario, the allocation of resources should prioritize interventions with a greater certainty of benefit, such as the use of personal protective equipment, vaccines, interventions for the ventilatory support of patients and pharmacological therapies with proven effectiveness. The treatment of patients should be encouraged through research protocols with an adequate design and potential to respond to society’s needs.

In addition to the evidence available in the scientific literature, the recommendations contained in the present updated guidelines considered aspects relevant in the Brazilian context, such as the availability of drugs in the country (whether due to regulatory or accessibility factors), the acceptability of interventions to patients and health professionals, and the costs associated with their use. Thus, these recommendations are applicable to both the Unified Health System (SUS - *Sistema Único de Saúde*) and supplementary health services. Additionally, most of the recommendations in this updated document are aligned with therapeutic approaches recommended to date by major international organizations and societies, such as the WHO, NICE, NIH, IDSA and SCCM.^(5,10,13,14)^

The present document consists of a joint positioning of seven medical societies, given the need to develop updated recommendations in a comprehensive manner and to contextualize them within different specialties in the face of the weaknesses of the available evidence and the relevance of the topic. Despite changes in the recommendations on the use of anticoagulants and tocilizumab, the inclusion of the recommendation on the use of baricitinib and the exclusion of the recommendation on the use of casirivimab + imdevimab, most of the recommendations of the first version of this guideline were not changed. This suggests a possible consolidation of guidelines for the pharmacological treatment of hospitalized patients with COVID-19. With these recommendations, we hope to provide national guidance for clinical practice related to pharmacological treatment for patients hospitalized with COVID-19, with the aim of promoting appropriate treatment and reducing the variability in the procedures applied.
